# Aggregates of Cyanine Dyes: When Molecular Vibrations and Electrostatic Screening Make the Difference

**DOI:** 10.1021/acs.jpcc.3c01253

**Published:** 2023-05-18

**Authors:** Francesco Bertocchi, Andrea Delledonne, Guillem Vargas-Nadal, Francesca Terenziani, Anna Painelli, Cristina Sissa

**Affiliations:** Dipartimento di Scienze Chimiche, della Vita e della Sostenibilità Ambientale, Università di Parma, Parco Area delle Scienze 17A, 43124, Parma, Italy

## Abstract

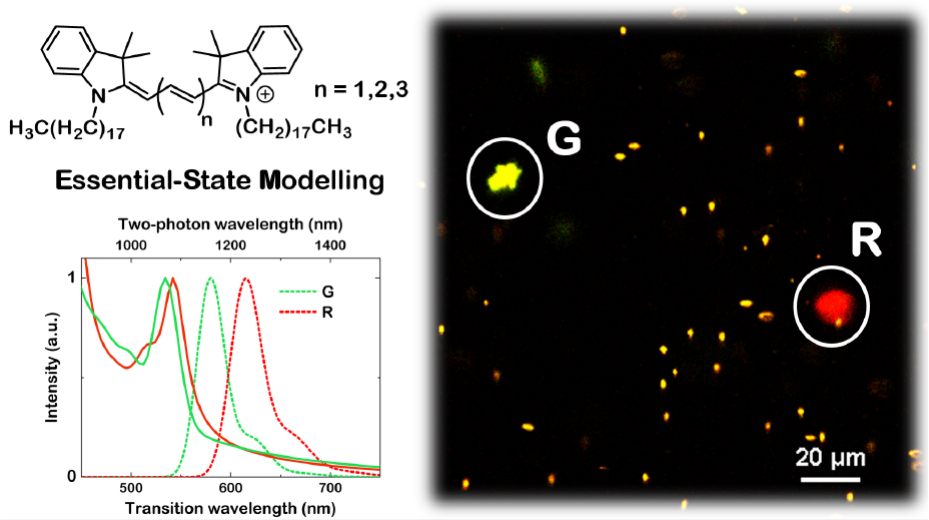

Aggregates of cyanine dyes are currently investigated as promising materials for advanced electronic and photonic applications. The spectral properties of aggregates of cyanine dyes can be tuned by altering the supramolecular packing, which is affected by the length of the dye, the presence of alkyl chains, or the nature of the counterions. In this work, we present a joint experimental and theoretical study of a family of cyanine dyes forming aggregates of different types according to the length of the polymethinic chain. Linear and nonlinear optical spectra of aggregates are rationalized here in terms of an essential-state model accounting for intermolecular interactions together with the molecular polarizability and vibronic coupling. A strategy is implemented to properly account for screening effects, distinguishing between electrostatic intermolecular interactions relevant to the ground state (mean-field effect) and the interactions relevant to the excited states (excitonic effects). To the best of our knowledge, this is the first attempt to simulate nonlinear spectral properties of aggregates of symmetric dyes accounting for molecular vibrations.

## Introduction

Cyanines are a widespread family of dyes, constituted by heterocyclic electron donors or acceptor groups linked through a polymethinic bridge.^1,2^ Cyanines are of interest for several applications, including photovoltaics,^3^ bioimaging,^4−7^ phototherapy,^8−10^ optical devices,^11,12^ sensors,^13^ etc. The tendency of cyanines to self-organize in aggregates is known since 1937 when Jelley and Scheibe first described the formation of cyanine aggregates in solution.^14,15^ The photophysics of cyanine aggregates strongly depends on the details of the molecular packing, which, in turn, are affected by several factors, including the length of the polymethinic bridge, the presence of non conjugated alkyl chains and their length, the environment (including the presence of additives), etc.^16−21^ The possibility to widely tune the material properties makes cyanine aggregates extremely promising for applications in photonics, electronics, imaging, etc.^22−26^ A robust theoretical approach must therefore be developed to relate the intriguing properties of cyanine aggregates to their supramolecular structure.

**Figure 1:**
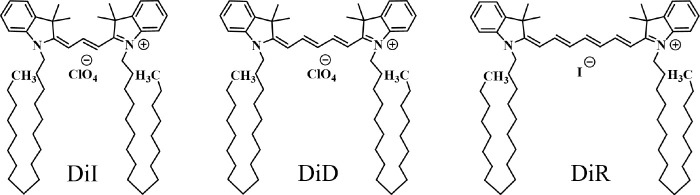
Molecular structures of the three cyanine dyes studied in this work.

Here, we present a joint experimental and theoretical work on self-assembled aggregates of DiI, DiD, and DiR (Figure 1), a family of cyanine dyes commercialized for fluorescence microscopy applications. The three molecules only differ in the length of the π-conjugated chain. An extensive spectroscopic study is carried out on the solvated dyes and on the aggregates in liquid suspension as well as embedded in a jelly matrix. In water/ethanol mixtures the dyes aggregate with important effects on linear (absorption and emission) and nonlinear (two-photon absorption) spectra. Specifically, we recognize the formation of J-aggregates for the shorter molecule DiI and of H-aggregates for DiD and DiR. This study offers a solid basis for a detailed theoretical analysis shedding light on the intertwined role of intermolecular interactions, molecular polarizability, vibronic effects, and environmental screening on the rich spectral properties of cyanine aggregates.

As for the theoretical modeling, we adopt a bottom-up strategy that relies on a comparatively simple and reliable model for the isolated dye in solution. An accurate description of low-energy excitations of cyanine dyes is challenging, and this class of molecules is often adopted to test the validity of theoretical approaches.^27−30^ Here we work in the spirit of essential-state models (ESM) and describe the low-energy physics of cyanine dyes in terms of few electronic basis (diabatic) states coupled to few effective molecular vibrations. The model, parametrized against experimental data, was successfully adopted to describe the photophysics of linear symmetric dyes,^31,32^ including cyanines.^33,34^ The model accounts for the nontrivial evolution of linear and nonlinear spectra with the solvent polarity, driven in cyanine dyes with long polymethinic chains by symmetry-breaking phenomena occurring in the ground state.

Once the ESM for the molecular unit is defined and accurately parametrized against experimental data, we build the aggregate model, introducing intermolecular electrostatic interactions. ESMs have been successfully applied to several types of molecular aggregates^35−40^ and have been recently adopted to investigate the reliability of the exciton approximation in aggregates of polarizable dyes with either a nonpolar or polar structure.^41,42^

Since cyanine aggregates are formed in highly polar environments (e.g., water/alcohols mixtures), special care is needed to properly address the screening of electrostatic intermolecular interactions. Polar solvents are characterized by high dielectric constants, that effectively screen the interactions between static charges. On the other hand, excitonic interactions are related to transition dipole moments (or more generally to transition charge distributions) that oscillate at optical frequencies. Accordingly, these latter interactions must be screened by the dielectric constant at optical frequency (i.e., the squared refractive index). Two different screening regimes must therefore be considered, as already discussed in a previous work on dimers of quadrupolar dyes bearing positive charges.^43^ Here, in an effort to also address nonlinear optical spectra of aggregates of cyanine dyes, we extend the model to account for molecular vibrations.

In the present work, the detailed spectroscopic characterization of DiI, DiD, and DiR in solution is described, with a discussion of UV–vis, fluorescence, and two-photon absorption spectra. The preparation procedure and the linear and nonlinear spectroscopic characterization of the aggregates are addressed. A detailed theoretical investigation of monomers and aggregates is presented, based on essential-state models. Finally, the results of modeling are compared with experimental data, emphasizing the nontrivial role played by molecular vibrations and electrostatic screening effects.

## Experimental Methods

### Materials

1,10-Dioctadecyl-3,3,30,30-tetramethyl-indocarbocyanine perchlorate (DiI), 1,10-dioctadecyl-3,3,30,30-tetramethyl-indodicarbocyanine perchlorate (DiD), and 1,1′-dioctadecyl-3,3,3′,3′-tetramethyl-indotricarbocyanine iodide (DiR) were purchased from ThermoFisher (Invitrogen). Agarose (low electroendosmosis, EEO ≤ 0.1) was obtained from Acros Organics. All chemicals were used without further purification. Spectra grade or HPLC solvents were used to prepare solutions. Bidistilled water was used to prepare aggregates.

### Linear Spectroscopic Characterization

UV–vis absorption spectra were recorded with a PerkinElmer Lambda650 spectrophotometer. Fluorescence spectra were collected with a FLS1000 Edinburgh Fluorimeter equipped with a R5509–72 (Hamamatsu) NIR-PMT for detection in the near-infrared region. The resulting intensity profiles were corrected for the excitation intensity and the detector sensitivity. For solutions/suspensions with absorbance higher than 0.1, fluorescence spectra were collected with a cuvette having optical path of 1.5 mm to minimize inner filter effects.

Fluorescence quantum yields and lifetimes for DiI and DiD in EtOH were taken from ref.^44^ In the case of DiR in EtOH, the fluorescence quantum yield was estimated using HITC in EtOH as a reference (Φ_*f*_ = 0.28 from ref (45)), employing the NIR-PMT detector to collect emission spectra of both DiR and reference.

Lifetime decay of DiR in ethanol has been collected exciting the sample with a pulsed diode laser (pulse duration < 200 ps) at a repetition rate of 1 MHz, exciting at 405 nm and collecting the emission at 780 nm.

### Dynamic Light Scattering Measurements (DLS)

The instrument employed for DLS measurements was a Malvern Zetasizer Nano ZSP, equipped with a He–Ne laser (633 nm). Intensity and volume distributions were obtained from the autocorrelation function of the scattered laser light at 173° (backscattering mode). All the suspensions were analyzed at 25 °C, and the results were averaged over three repetitions.

### Nonlinear Spectroscopic Characterization

Two-photon excited measurements were carried out using a Nikon A1R MP+ multiphoton upright microscope. A tunable (700–1300 nm) femtosecond mode-locked laser (Coherent Chameleon Discovery) was used as radiation source and a water dipping objective (25×, NA = 1.1) was employed to focus the excitation beam and to collect the outcoming two-photon excited fluorescence (TPEF) signal. The TPEF signal was then detected by three nondescanned detectors (NDDs) and a spectral detector connected to the microscope through an optical fiber. The series of NDDs is composed of two high sensitivity Gallium Arsenide Phosphide (GaAsP) and a Multi-Alkali photomultiplier tubes (PMTs), each one preceded by a specific filter cube in order to detect different spectral regions: green (506–593 nm) and red (604–679 nm) for the GaAsP’s PMTs and far-red (698–750 nm) for the Multi-Alkali PMT. The spectral detector is a GaAsP PMT preceded by a dispersive element that allows to collect emission spectra in the 400–650 nm region with a 10 nm resolution. When using the NDDs, the associated dichroic filter allows for excitation in between 820 and 1300 nm, while the spectral detector is associated with a dichroic mirror allowing for excitation in the 700–1080 nm region. The TPEF images were recorded using the three NDDs, merging the three simultaneously acquired channels through the operation software of the microscope.

The two-photon absorption (TPA) cross section σ_2_, which expresses the probability of the TPA process, can be measured using a relative method. Fluorescein in NaOH 0.1 M was used as a reference (Φ_*f*,*ref*_ = 0.9), considering the absolute values of cross section reported in the literature.^46^ The following equation was used to estimate the sample’s cross section as a function of the incoming wavelength λ:^47^
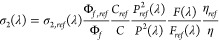
1where Φ_*f*_ is the fluorescence quantum yield, *C* is the solution concentration, *F* is the integrated TPEF spectrum, and *P* is the laser power and η the refractive index of the solvent. The subscript “*ref*” indicates the properties of the reference, while all the others terms refer to the sample. The reliable comparison of the integrated TPEF spectra of sample (*F*) and reference (*F*_*ref*_) requires to collect the major part of the TPEF spectra and correct them for the wavelength-dependent sensitivity of the employed detector. This is possible only for the spectral detector, so only for samples whose emission band mainly falls inside its sensitivity region (400–650 nm). Unfortunately, NDDs do not allow to correct the TPEF intensity (the signal collected by the NDDs is relevant to the whole bandwidth of the corresponding bandpass filter). The TPEF signal was measured with NDDs and/or with the spectral detector, according to the excitation and fluorescence spectral ranges of the sample. More specifically, in the case of DiI and DiD, for excitation between 700 and 1080 nm, the TPEF is detected using the spectral detector (for DiD only a tiny portion of the emission spectrum falls inside the 400–650 region, so that a TPEF signal could be measured with the spectral detector but a scale factor accounting for the missing part of the emission spectrum, needed to estimate the cross section, could not be reliably guessed). For excitation above 820 nm, TPEF is detected using the green (for DiI) and the red (for DiD) NDDs. The overlapping excitation region (820–1080 nm) was used to merge the two parts of the excitation spectra acquired with different detectors. For DiR in solution, only the far-red NDD is suitable for the detection of its TPEF signal and the sample was excited in the 900–1300 nm spectral region. For the explained reasons, the TPA cross section could not be quantified for DiD and DiR, whose fluorescence (or most of it) falls outside the region covered by the spectral detector: for these compounds, only the band shapes of the corresponding TPA spectra are available. TPEF of DiI, instead, could be (almost entirely) measured with the spectral detector, so that the TPA cross section could be retrieved for this sample. As TPA involves the simultaneous absorption of two photons, its probability should be quadratic with the excitation power. For TPA spectra collected in solution, deviations from quadraticity resulted below ±15% repeating the measurements with three different laser powers. For aggregates in suspension, the deviation from quadraticity resulted to be more critical (up to ±25% in a few points) due to the intrinsic nonhomogeneity of the sample. For TPA spectra of aggregates in the gel, the quadraticity was not tested to prevent photobleaching of the sample.

Liquid samples (solutions and suspensions) were analyzed in quartz cuvettes placed horizontally under the microscope objective. Each cuvette was completely filled with the liquid sample to avoid the presence of air between the upper wall and the solution. Distilled water was employed to ensure the contact between the objective and the cuvette. Each measurement has been conducted focusing the excitation beam as near as possible to the cuvette upper wall, to avoid artifacts due to the different refractive index of the solvent and inner-filter effects.

### Preparation of Aggregates

#### Aggregates in Water/Ethanol Suspension

##### DiI Aggregates

65 mg of a previously sonicated 1220 μM stock solution of DiI in ethanol was put into a dark vial. Ethanol was added until reaching 1.5 g of mass, followed by bidistilled water being rapidly added at room temperature until the mixture was 5 g in weight, in order to obtain a 70/30 m/m mixture of water/ethanol. The resulting mixture was homogenized at the vortex for 40 s.

##### DiD Aggregates

149 mg of a previously sonicated 530 μM stock solution of DiD in ethanol was put into a dark vial. Ethanol was added until reaching 0.5 g of mass, and then bidistilled water was rapidly added until the mixture was 5 g in weight, in order to obtain a 90/10 m/m solution of water/ethanol. The resulting mixture was homogenized at the vortex for 40 s.

##### DiR Aggregates

41 mg of a a previously sonicated 1970 μM stock solution of DiR in ethanol was put into a dark vial. Ethanol was added until reaching 0.5 g of mass, and then bidistilled water was rapidly added until the mixture was 5 g in weight, to obtain a 90/10 m/m solution of water/ethanol. The resulting mixture was homogenized at the vortex for 40 s.

##### Aggregates in the Gel

0.5 g of agarose powder was weighed in a 50 mL beaker, and 25 mL of bidistilled water was added, forming a white suspension. The suspension was heated up to its boiling point. After boiling for 20 min, the agarose powder was completely dissolved, and a water-clear solution was obtained. The agarose solution was cooled down until it reached 40 °C, then 1 mL of aggregates suspension in water/ethanol mixture was added. The resulting suspension, containing the agarose and the aggregates, was poured in a plastic cuvette to register UV–vis absorption and in a small circular plastic holder for linear fluorescence and two-photon excited microspectroscopy. We point out that at 40 °C the suspension is still liquid, and the hydrogel is then obtained after cooling down at room temperature. The temperature at which aggregates are added is crucial: if it is too high, then they could break or modify; if it is too low, then they would not diffuse homogeneously into the bulk due to its high viscosity. In order to verify that aggregates are not significantly damaged in the process, absorption, and emission of the dye-containing gel were acquired (Figure S7).

## Results and Discussion

### Spectroscopic Characterization

#### Cyanine Dyes in Solution

**Figure 2:**
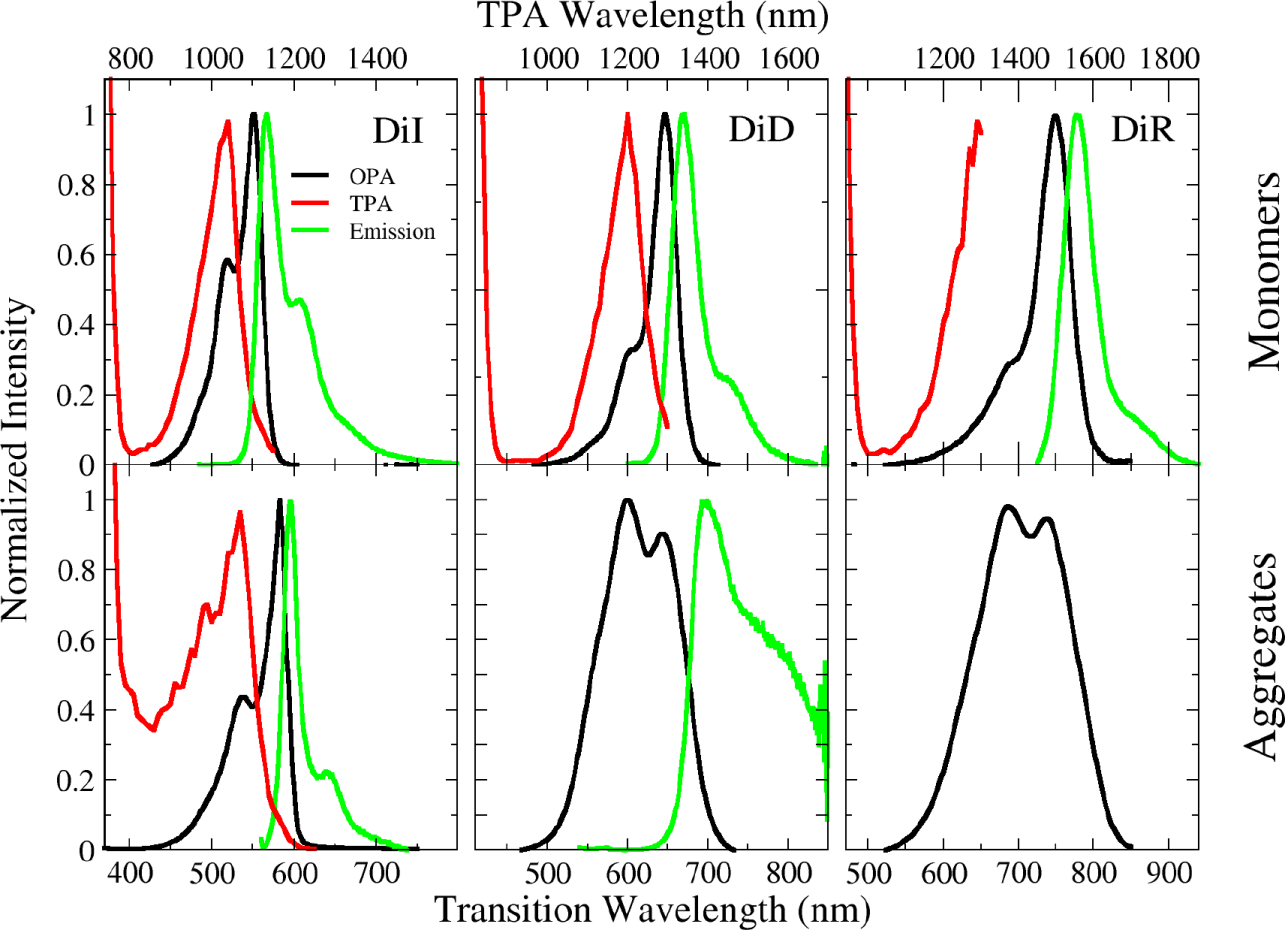
OPA (black lines), TPA (red lines), and emission (green lines) spectra of DiI, DiD, and DiR monomers in ethanol (top panels) and aggregates in water/ethanol mixtures (bottom panels).

One-Photon Absorption (OPA), emission, and Two-Photon Absorption (TPA) spectra of DiI, DiD, and DiR dissolved in ethanol (a good solvent for cyanine dyes)^48^ are reported in Figure 2. All spectra move to the red upon increasing the length of the polymethinic bridge, in line with the increased delocalization length. At the same time, the relative intensity of the 0–1 vibronic transition progressively decreases as the length of the molecule increases, both in absorption and in emission, an indication that the equilibrium geometries of the ground and first excited state become more similar for longer cyanines. The large molar extinction coefficients (Table 1) are typical of cyanine dyes and are related again to the delocalization of electrons involved in the transition. Fluorescence quantum yields are high, particularly with reference to the emission spectral region which goes from yellow (DiI) to red (DiD) to far-red (DiR). Fluorescence lifetimes are in the nanosecond range, as reported in Table S1. OPA and emission spectra are mirror images, and the Stokes shifts are negligible (Table 1), suggesting minor structural and solvent reorganization after excitation. Accordingly, marginal effects of polar solvation are expected, as confirmed by the negligible dependence of absorption and emission spectra on the solvent polarity (Figure S1).

**Table 1:** Spectroscopic Data of Cyanine Dyes Dissolved in Ethanol^a^

	λ_*max*_^*abs*^ (nm)	λ_*max*_^*em*^ (nm)	ϵ_*max*_ (L mol^–1^ cm^–1^)	Φ_*f*_	Stokes shift (cm^–1^)
DiI	550	567	140000^b^	0.1^b^	487
DiD	647	670	246000^b^	0.37^b^	511
DiR	750	780	305000	0.37	513

a Φ_*f*_ indicates the fluorescence quantum yield.

b From ref (44).

TPA spectra of the three dyes in ethanol solution (Figure 2) were collected with a multiphoton microscope, measuring the two-photon excited fluorescence (TPEF, technical details are reported in the “Experimental Methods” section). TPA has different selection rules with respect to OPA, and specifically, symmetric (*gerade*) states, that are OPA forbidden, are bright in TPA. The TPA spectra of the three dyes show the tail of an intense band (350–450 nm transition wavelength, see also Figure S2) whose maximum is not accessible with our experimental setup. In this spectral region, the OPA intensity is negligible, suggesting that this state is described by a symmetric (*gerade*) wave function. However, in the region where the OPA band is observed, a weak TPA signal is collected, whose maximum is located at the same frequency as the 0–1 vibronic transition of OPA (due to the setup limitation, the maximum of this TPA band is not accessible for DiR). The TPA cross section was measured only for DiI (Figure S3), amounting to σ_2_ = 46 GM (Goeppert–Mayer, 1 GM = 10^–50^ cm^4^ s photon^–1^) at 520 nm. The experimental setup for measuring TPA spectra is not suitable for the measurement of the cross section of DiD and DiR (more details are reported in the “Experimental Methods” section).

##### Cyanine Aggregates

Aggregates of the three dyes were prepared in water/ethanol mixtures, as described in the “Experimental Methods” section. Aggregation is confirmed by dynamic light scattering (DLS) measurements, as reported in Table S3. The suspensions of DiI and DiR aggregates show a bimodal size distribution, with average dimensions of ∼73 nm and ∼83 nm, respectively (additional details about DLS are reported in Table S3 and Figure S4). A single population of nanoparticles is detected for DiD, with an average hydrodynamic diameter of ∼38 nm.

OPA, emission, and TPA spectra of aggregates are shown in the bottom panels of Figure 2 and spectroscopic data are summarized in Table 2. OPA and emission spectra of DiI are clearly consistent with J-aggregation: both bands are shifted to the red if compared to the monomer, the Stokes shift is marginal, and the ratio of the 0–1 vs the 0–0 vibronic band decreases, pointing to an exciton delocalization length ∼2.^49,50^ On the opposite, DiD and DiR spectra suggest H-aggregation: the OPA band is broader and blue-shifted vs the monomer band and fluorescence is suppressed. Indeed DiR fluorescence was not detected, while a weak emission is observed for DiD, largely red-shifted with respect to OPA. This behavior is consistent with the observation of a vibronically induced fluorescence from H-aggregates.^41,51^ The TPEF technique used to collect TPA spectra only works for emissive species, so we were able to obtain data only for DiI aggregates (DiD aggregates emission is too weak). Much as with monomers, TPA spectra of DiI aggregates are blue-shifted compared to OPA, with the TPA maximum located at the frequency of the 0–1 vibronic band of the OPA spectrum of the aggregates.

**Table 2:** Spectroscopic and DLS Data of Cyanine Aggregates in Water/Ethanol Mixtures^a^

aggregates	λ_*max*_^*abs*^ (nm)	λ_*max*_^*em*^ (nm)	Stokes shift (cm^–1^)	*Z*-average (nm)
DiI	586	596	278	83.1
DiD	600	700	2381	37.8
DiR	690	n.d.	n.d.	72.8

a *Z*-average is the average hydrodynamic diameter of the nanoparticles.

Absorption spectra of DiI aggregates were collected as a function of temperature (Figure S5). After the first temperature cycle, absorption changes significantly with respect to spectra collected just after preparation, suggesting that the prompt formation of aggregates is kinetically driven, while thermal treatment allows for the formation of thermodynamically favored aggregates. This is supported by the observation that absorption does not vary after a second temperature ramp. For DiD aggregates, variations after heating are smaller and could be due to partial breaking of aggregates (Figure S6).

**Figure 3:**
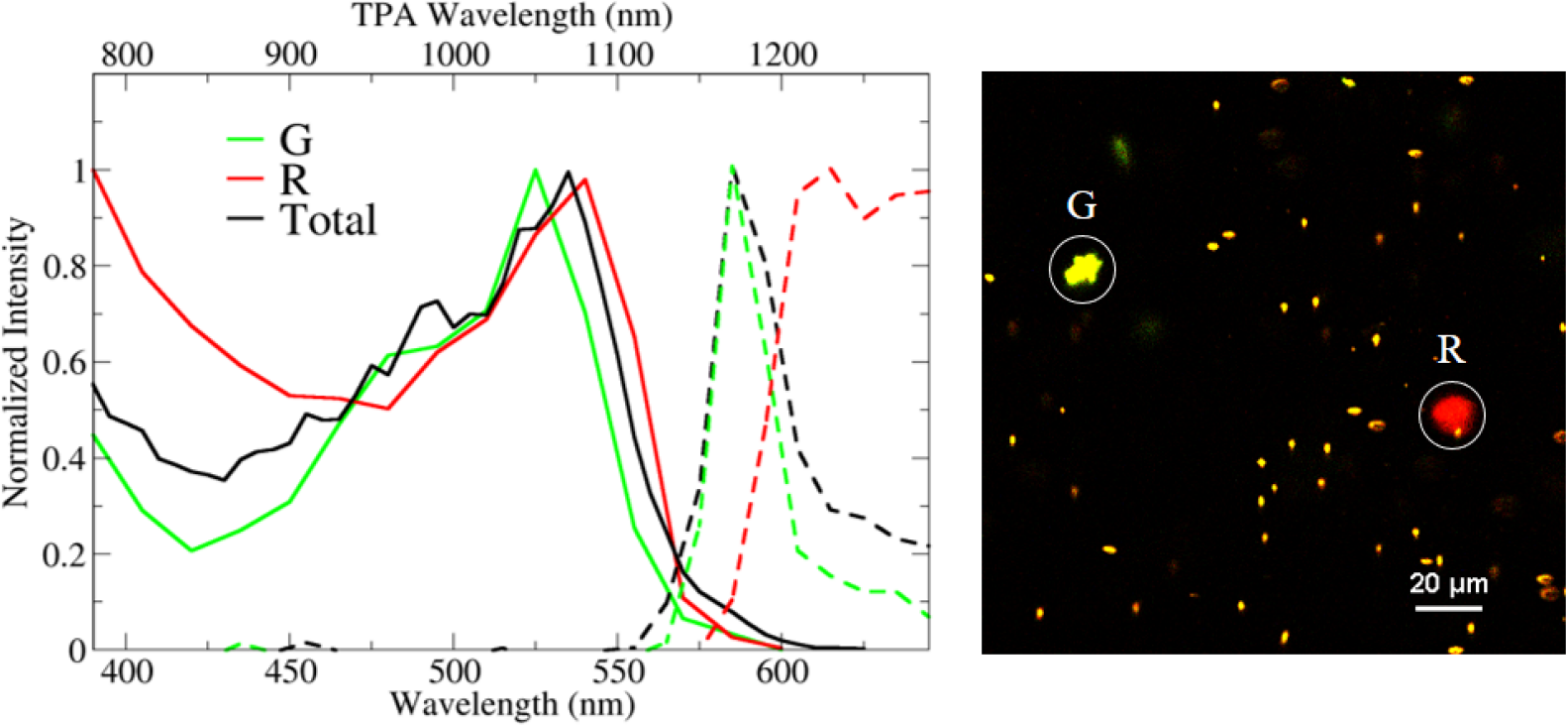
Left panel: two photon excited emissions (dashed lines) and excitation (continuous lines) spectra of single nanoparticles in the gel. Right panel: two-photon excited fluorescence image of the aggregates chosen for single aggregate spectra. Additional images of aggregates are reported in ESI (Figure S8).

The good fluorescence of DiI allowed for the microscopic characterization of aggregates with the two-photon microscope. To such an aim, DiI aggregates were dispersed in an agarose hydrogel (see the “Experimental Methods” section for the preparation procedure), a highly viscous medium that hinders the diffusion of nanoparticles during the measurement. In this way, we collected images and spectra from single (immobilized) aggregates *t*. First, we verified that OPA, emission, and TPA spectra collected from the hydrogel coincide with the ones collected in suspension (Figure S7), thus confirming that the hydrogel environment does not significantly affect the aggregate spectroscopic behavior. Figure 3 shows a TPEF image of DiI aggregates collected with the two-photon microscope. Aggregates of different sizes and different colors are imaged, in line with DLS data that point to polydisperse suspensions. The two bigger aggregates, labeled as “G” and “R” in Figure 3, were selected to collect single-aggregate fluorescence and TPA spectra, shown in the left panel of Figure 3. Aggregate “G” is greenish, with an emission spectrum peaked at 585 nm, almost overlapping the spectrum collected on the bulk hydrogel or equivalently the spectrum collected from the liquid suspension. Aggregate “R” shows weak red fluorescence, as typical of H-aggregates,^41^ with a broad spectrum, extending from ∼600 nm downward, outside of the region accessible with the spectral detector coupled to the microscope. The TPA bandshapes of the two aggregates are very similar, at least within the 10 nm spectral resolution of the setup. The TPA spectrum of the “R” aggregate is slightly red-shifted with respect to the green aggregate, and the total TPA spectrum of the hydrogel is intermediate between them. We conclude that DiI forms both H- and J-aggregates with distinctively different fluorescence spectra, and the overall emission of the suspension and of the gel is largely dominated by the fluorescence of J-aggregates, which is much more intense than the emission from H-aggregates.

### Essential-State Models for Monomers and Aggregates

#### Three-State Model for Monomers

To describe the low-energy physics of the three cyanine dyes of interest, we adopt a three-state model originally developed for linear quadrupolar dyes,^31^ and then extended to cyanine dyes.^33,34^ For the sake of clarity, here we just outline the model, but more details can be found in the Supporting Information and in the original papers.^31,33,34^

**Figure 4:**
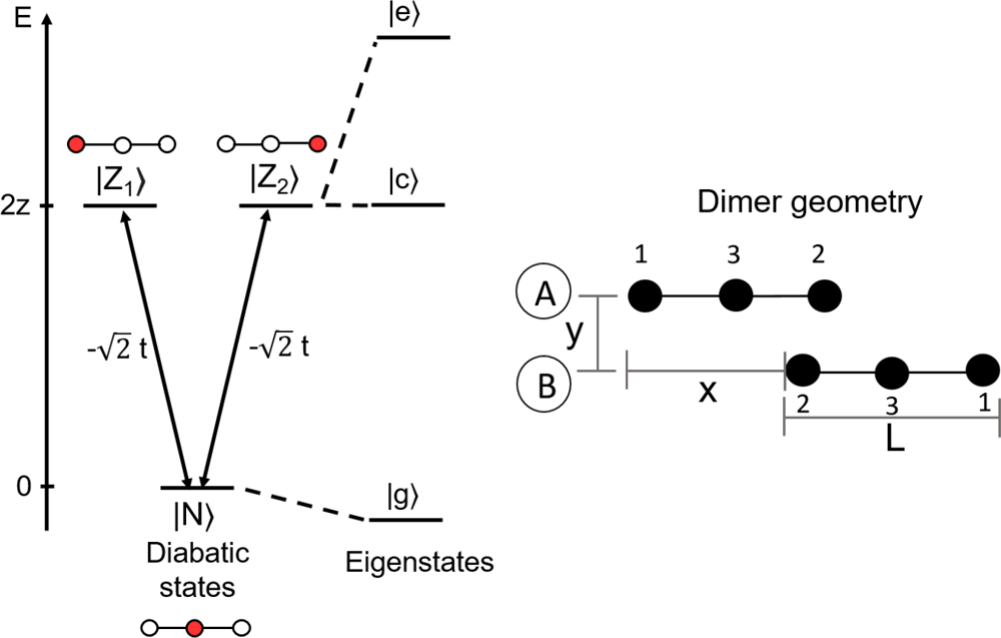
Left: scheme of diabatic basis states |*N*⟩, |*Z*_1_⟩, |*Z*_2_⟩ and of the eigenstates |*g*⟩, |*c*⟩, |*e*⟩ of a cyanine monomer. The red filled circles show where the net charge is located in the basis states. Right: schematic representation of a cyanine dimer. The black filled circles represent the three sites, numbered as 1, 2, and 3, of the two molecules, labeled as A and B. *L*, *x*, and *y* are the geometrical parameters that define the arrangement of the two molecules in the dimer.

As sketched in Figure 4, we consider three molecular sites, 1 and 2 corresponding to the two terminal electron donor (D) sites and 3 corresponding to the polymethinic bridge, π. The electronic structure is then minimally described by three basis states, corresponding to the three main resonance structures: |*N*⟩ represents the state D−π^+^–D, while  and  represent D–^+^π–D and D−π–D^+^, respectively. The energy of the |*N*⟩ state is set to 0, while the two degenerate states  and  have energy 2*z*. Charge hopping is allowed from the central bridge to the lateral groups, with  measuring the matrix element mixing both  and  with |*N*⟩. The dipole moment of a charged object is not defined, but to address spectral properties, we define an effective dipole moment operator, measuring the charge unbalance of  and  with respect to |*N*⟩. Accordingly, the nonvanishing matrix elements of the dipole moment operator in the chosen diabatic basis are  and  (the relevant component of the dipole moment is parallel to the main molecular axis). In symmetric dyes, the two degenerate basis states  and  are conveniently combined in symmetric and antisymmetric states: . The symmetric |*N*⟩ and  states mix to give the two symmetric eigenstates: the ground state |*g*⟩ and the excited state |*e*⟩. The antisymmetric  state stays unmixed and coincides with the |*c*⟩ eigenstate. Due to the mixing between |*N*⟩ and , in the ground state the charge distribution on the three sites of the molecule can be described in terms of the parameter ρ, that accounts for the charge displacement from the central site to the two lateral sites of the molecule (the molecule is symmetric, and the charge is equally distributed on lateral sites). Since the total charge is +1, the central site (corresponding to the π bridge) bears a positive charge of + (1 – ρ). The overall charge distribution in the ground state is D^+ρ/2^π^+(1−ρ)^D^+ρ/2^ (see the Supporting Information). The symmetric |*e*⟩ state, forbidden in OPA, is responsible for the intense TPA band observed at transition wavelengths shorter than 500 nm, as shown in Figure 2 (the maximum of these bands are not accessible by our experimental setup).

To address spectral bandshapes and, when relevant, symmetry-breaking phenomena, the model was extended to account for electron-vibration coupling.^31,34^ To such an aim, two effective harmonic vibrational coordinates, *q*_1_ and *q*_2_, are introduced to describe the rearrangements of the molecular geometry upon charge redistribution from |*N*⟩ to  and , respectively. The two vibrational modes are equivalent, with harmonic frequency ω_*v*_ and relaxation energy ε_*v*_. The vibronic Hamiltonian for the monomer reads:
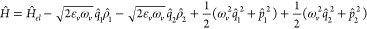
2where  is the three-state electronic Hamiltonian described above (see also eq S5),  and  are the operators that measure the charge on the two lateral sites in the cyanine. The electron-vibration problem is solved numerically, fully accounting for the nonadiabatic nature of the coupling (see the Supporting Information for technical details). The numerically exact eigenstates of the molecular Hamiltonian finally enter the calculation of absorption, emission, and TPA spectra, as described in ref (52) and summarized in the Supporting Information. Polar solvation has marginal effects in these cyanine dyes, as demonstrated by negligible absorption and emission solvatochromism (Figure S1), and will be disregarded.

**Figure 5:**
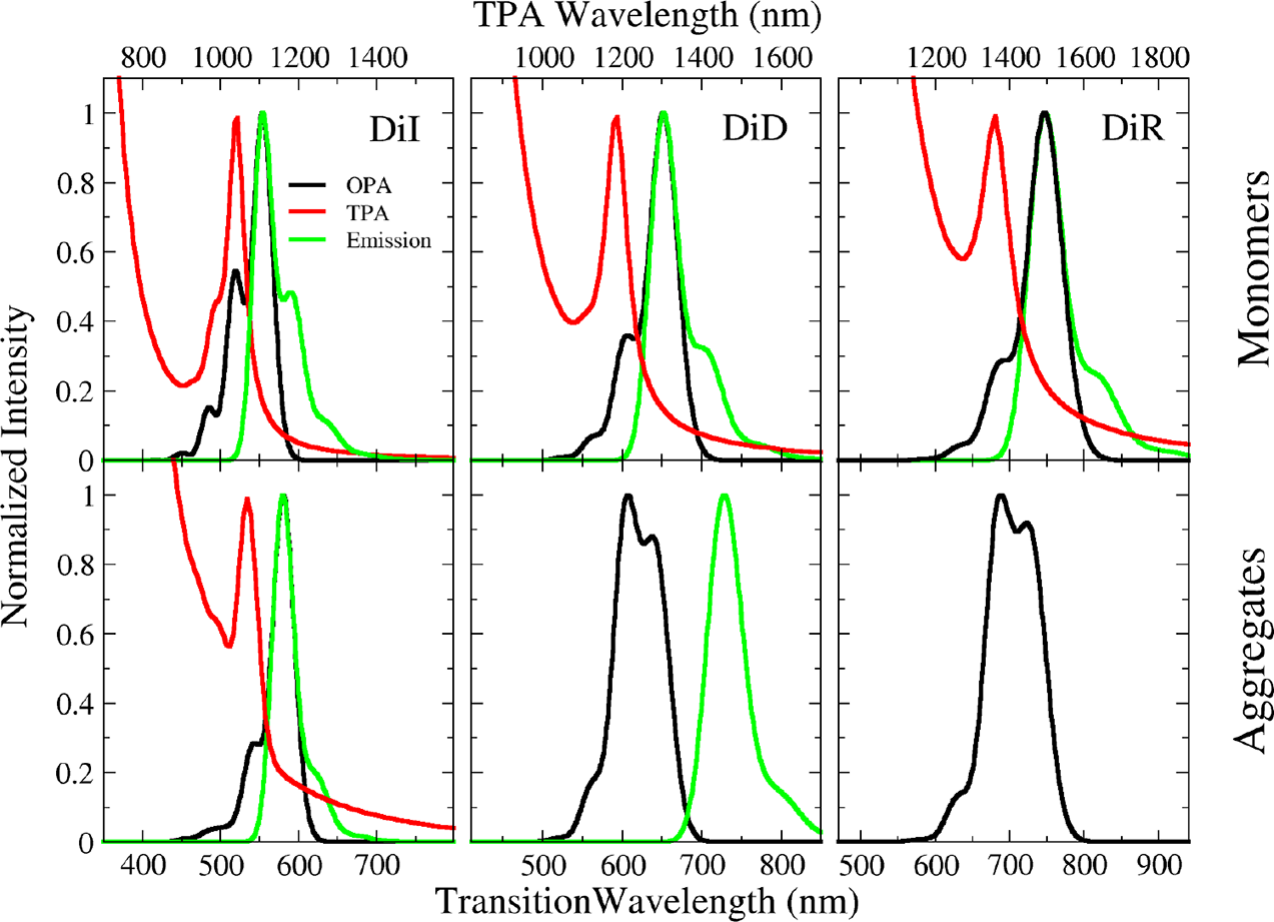
Calculated OPA (black lines), TPA (red lines), and emission (green lines) spectra of DiI, DiD, and DiR monomers (top panels) and dimers (bottom panels). Geometrical parameters for the dimers: *L* = 7 Å, *x* = 7 Å, *y* = 3 Å for DiI; *L* = 8 Å, *x* = 3 Å, *y* = 6.8 Å for DiD; *L* = 9 Å, *x* = 4 Å, *y* = 6 Å for DiR (the definition of geometrical parameters is reported in Figure 4). Calculations are performed adopting static (ϵ) and optical (η^2^) dielectric constants of water, ϵ = 78 and η^2^=1.8 respectively.

The proposed model is semiempirical in nature and model parameters in Table 3 are selected to best reproduce experimental spectra. It is worth mentioning that, for each dye, only six molecular parameters are required to describe the position and band shapes of OPA, TPA, and emission spectra. Indeed μ_0_ does not affect spectral band shapes but only their intensity, so that for each dye μ_0_ was set to the value needed to reproduce the molar extinction coefficient. With this choice, the TPA cross section of DiI is estimated as 47 GM at 520 nm, in very good agreement with the experimental data. Overall, calculated spectra in Figure 5 reproduce well experimental data in Figure 2, in terms of spectral position and band shapes, including the vibronic progression. This result confirms that the three-state model captures the most important spectral features of the cyanine molecules under investigation.

**Table 3:** Set of Parameters Used to Fit Cyanine Monomer Spectra

cyanine	*z* (eV)	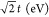	ω_*v*_ (eV)	ϵ_*v*_ (eV)	γ (eV)	μ_0_ (D)
DiI	0.105	1.689	0.15	0.745	0.06	19.1
DiD	0.11	1.4	0.15	0.4	0.06	26.4
DiR	0.15	1.15	0.15	0.32	0.06	33.0

Looking at the spectra in more detail, the TPA transition toward the lowest state (state “0”) of the vibrational manifold relevant to the lowest excited electronic state is forbidden, while the transition toward the successive vibrational state (state “1”) acquires sizable intensity, in agreement with experimental data. In centrosymmetric molecules, allowed TPA states must be symmetric. In our model, the lowest electronic excited state, |*c*⟩, is antisymmetric, and as a consequence, the electronic contribution to the TPA intensity is vanishing (in other words, the |*c*⟩ state is forbidden in TPA). The TPA intensity becomes sizable thanks to the coupling with antisymmetric vibrations (Herzberg–Teller effect). For the sake of clarity, in the framework of this discussion it is useful to adopt the Born–Oppenheimer (or adiabatic) approximation: the vibronic wave function is the product between an electronic and a vibrational function. In our model, the two molecular vibrations (*q*_1_ and *q*_2_) recombine in a symmetric and an antisymmetric mode, . For an electronic antisymmetric state (|*c*⟩), the total vibronic wave function is overall symmetric only if the vibrational part of the wave function is antisymmetric: this only occurs for the odd vibrational states related to the antisymmetric coordinate *q*_–_. In summary, the formally TPA forbidden transition toward the |*c*⟩ state acquires intensity thanks to the coupling with the antisymmetric vibrational coordinate. In particular, while the 0–0 TPA transition is symmetry-forbidden, the 0–1 transition is symmetry-allowed (its intensity is low because it is related to small vibrational displacements around the equilibrium geometry).

#### Modeling Cyanine Aggregates

Modeling aggregates is a complex issue in several respects. First of all, it is hard to obtain reliable information on the precise supramolecular arrangement of the dyes in the aggregate. Moreover, molecular vibrations enter the problem nonadiabatically,^41^ so that addressing large aggregates becomes extremely demanding, because very large basis are required. In the following, we will limit our discussion to cyanine dimers, as to maintain a reasonable dimension of the problem, while acquiring a good understanding of the complex physics of the aggregates. Experimental spectroscopic data on DiI aggregates show that the 0–0 vibronic transition, allowed in OPA, is forbidden in TPA, much as observed for the DiI monomer in ethanol. This suggests that the aggregate maintains a centrosymmetric structure as to support the mutual exclusion rule. Therefore, we will consider simple centrosymmetric cyanine dimers as illustrated in the right panel of Figure 4. The geometry of the dimer is defined by the intermolecular distance, *y* in the figure, and by the offset, *x*. The effective length of the chromophoric core *L* is set to 7, 8, and 9 Å for DiI, DiD, and DiR, respectively.

The physics of aggregates is driven by intermolecular electrostatic interactions. As discussed in recent literature,^36−38,42,43,53−56^ essential-state models lend themselves quite naturally to introduce intermolecular interactions. The diabatic basis set for the dimer is the direct product of electronic basis states  and  of the monomer (see Table S4). On this basis, intermolecular electrostatic interactions are diagonal and can be easily estimated from the aggregate geometry. Specifically, the dimer Hamiltonian reads:
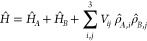
3where  are the molecular Hamiltonians for the two isolated dyes, labeled as in Figure 4. The third term accounts for intermolecular interactions, with  measuring the amount of charge on the *i*-th site of molecule A/B (3 being the central site, ), while *V*_*ij*_ measures the repulsion energy between two unit charges on sites *i* and *j* located on different molecules (explicit expressions for *V*_*ij*_ are reported in eqs 9 in the Supporting Information).

There is however an issue, related to the screening of the interactions. In our case, aggregates are formed in water/ethanol mixtures. The two solvents are highly polar (static dielectric constants are 80 and 24.5 for water and ethanol, respectively). The mixture then constitutes a highly polar environment: the large dielectric constants of both solvents (and hence of their mixture) produce a large screening of static interactions. On the opposite, the dielectric screening at optical frequencies, as measured by the squared refractive index, amounts to ∼1.8, a typical value for common organic solvents (water and ethanol refractive indices are 1.33 and 1.36, respectively). Therefore, interactions between static charges should be largely screened by the static dielectric constant, while the screening related to oscillating dipoles should be much smaller, being related to the squared refractive index. The delicate issue is how to discriminate the two kinds of interactions. Indeed this is not possible by adopting the diabatic basis, since the third term of eq 3 accounts for static and dynamical interactions at the same time.^42,43,53,56^

Following an approach developed some years ago for aggregates of quadrupolar dyes,^43^ a step-by-step procedure is adopted. First, a mean-field Hamiltonian is defined to accurately describe the ground-state properties of the dyes inside the aggregate. At the mean-field level, only static (ground state) properties are addressed, and intermolecular electrostatic interactions are screened by the static dielectric constant, ϵ. In the second step, the eigenstates of the molecular mean-field Hamiltonian are used to rotate the Hamiltonian in the adiabatic (or exciton) basis, where the states are classified according to the number and type of excitation. Once the Hamiltonian is written on this basis, it is easy to single out excitonic interactions (i.e., interactions that only account for exciton migration).^53^ In line with the exciton model, we only consider interactions between degenerate states: these interactions are screened by the squared refractive index at optical frequency, η^2^. The effect of ultraexcitonic terms (i.e., off-diagonal terms mixing nondegenerate states) is small on both OPA and TPA and can be evaluated numerically as shown in Figure S11.

In the mean-field approach, the Hamiltonian of a single molecule is diagonalized accounting for the presence of the electrostatic potential generated by the other molecule(s). Indeed, positive charges on the sites of molecule B affect the energy required to locate charges in the different sites of molecule A, an effect that reflects on the renormalization of the *z* parameter. In turn, the renormalized *z* leads to a variation of the charge distribution in the molecule. Since the two molecules are equivalent, we force the same charge distribution in corresponding molecular sites (see Figure 4), leading to a self-consistent problem, that is numerically solved as schematically shown in Figure S10. We notice that in dimers with a finite offset (*x* ≠ 0 in Figure 4) the sites 1 and 2 in each molecule are no more equivalent, so that the two diabatic states  and  are no more degenerate.

**Figure 6:**
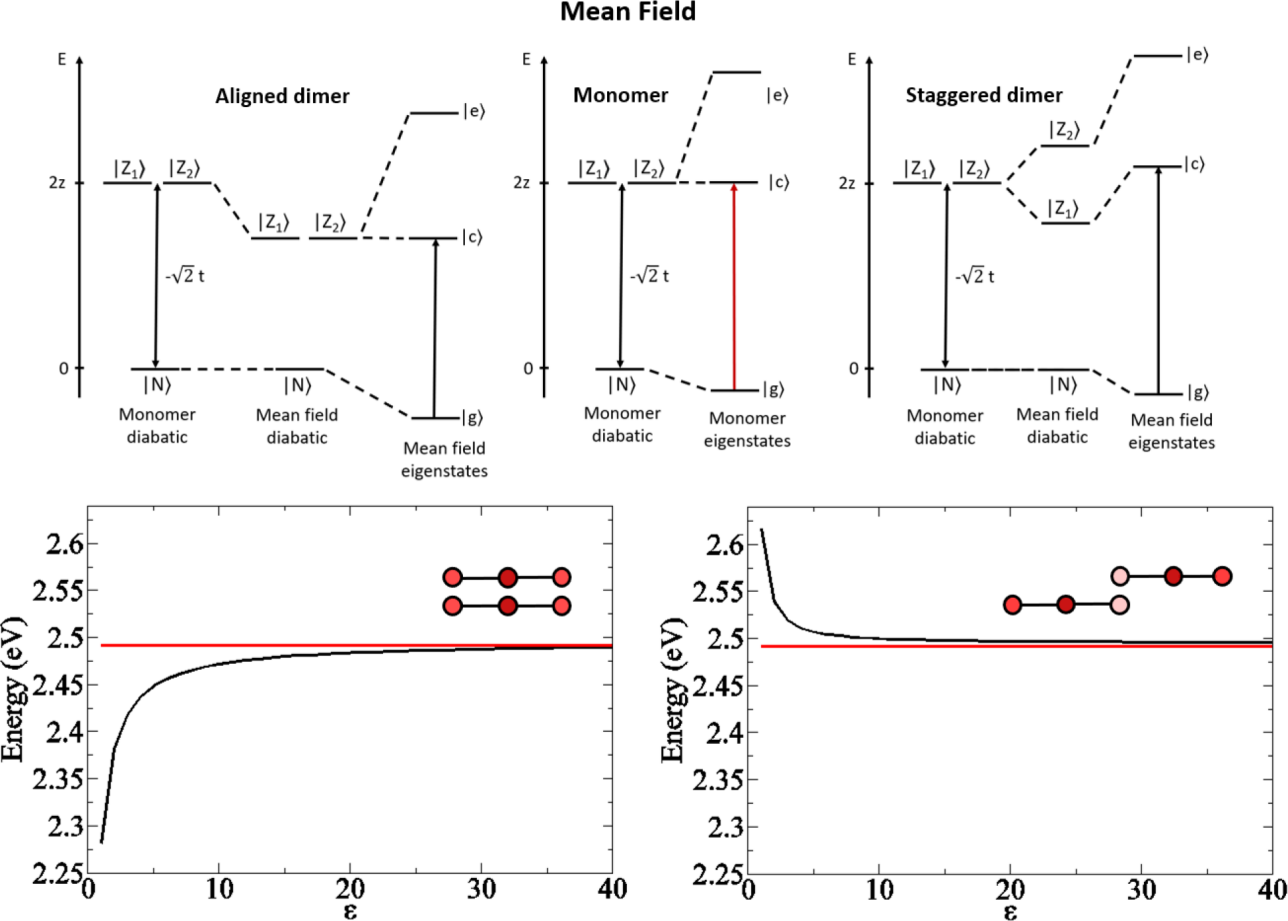
Top panels: schematic representation of mean-field approach to the dimers. The central panel refers to the isolated monomer (same information as in Figure 4). Left and right panels (referring to the aligned and staggered dimers, respectively) show how the energy of diabatic states is affected by the mean-field potential. In the lower panels, in a schematic representation of the two dimers, the intensity of the red color assigned to each site gives a qualitative representation of the amount of charge on the corresponding site, as calculated in mean-field approximation for the ground state. In the two graphs, the red line shows the OPA transition energy of the monomer, while the black line shows the lowest OPA transition energy of the dimer calculated as a function of the medium dielectric constant in the mean-field approximation.

We discuss mean-field effects considering two dimers with interplanar distance *y* = 4 Å: one in aligned geometry (*x* = 0) and one in staggered geometry *x* = 6 Å. Resulting mean-field charges on the molecular sites are reported in Table S5. Here we notice that, as schematically illustrated in Figure 6, in the aligned dimer, the charge flows from the bridge to the lateral sites as to minimize electrostatic repulsion. For the same reason, in the staggered dimer the charge moves toward the outer sides.

The bottom panels of Figure 6 show the OPA transition energies calculated in the mean-field approach for aligned (left) and staggered (right) DiI dimers as a function of the static dielectric constant. Mean-field effects are sizable in low-polarity environments (see also Table S6 where results on the mean-field charge distribution are listed for a system with ϵ = 5), while they become negligible in medium/high polarity environments. In our case, in water/ethanol mixtures with ϵ ∼ 60–70, mean-field effects are negligible for both geometries. We notice, however, that the aligned geometry (left-bottom panel of Figure 6) corresponds to an H-aggregate, where the exciton model predicts a blue-shift of the absorption band. However, mean-field effects lead to an opposite effect: a red-shift of the lowest transition with respect to the monomer is observed (for small values of the dielectric constant).^37,42,55,57−59^

**Figure 7:**
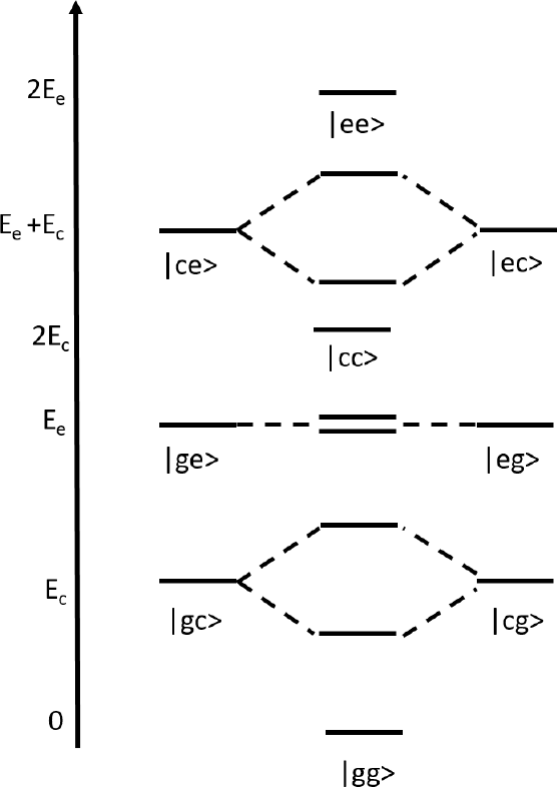
Schematic view of the exciton basis states and of their mixing as due to the interactions between degenerate states (the only interactions retained in the exciton approximation). The mixing matrix elements between |*ge*⟩ and |*eg*⟩ is very small leading to a small splitting, as detailed in the Supporting Information.

The mean-field adiabatic eigenstates are then used to build the exciton basis as the direct product of the three adiabatic eigenstates, |*g*⟩, |*c*⟩, |*e*⟩, for each monomer, for a grand total of 9 electronic states (see Table S7). Since the mean-field states are linear combinations of the basis states, it is possible to rotate the Hamitonian on the new exciton basis. For aggregates of polar dyes, where the molecular Hamiltonian is defined on just two states, an analytical transformation is possible.^42,53^ In our case, we resort to a numerical transformation, as explicitly addressed in the Supporting Information. Since several electrostatic interaction terms appear as a result of the transformation, we apply the exciton approximation disregarding all terms that mix nondegenerate states. The remaining exciton interaction terms, schematically illustrated in Figure 7, are screened by the squared refractive index of the medium.

We can validate the exciton approximation repeating the calculation for a dimer in a nonpolar environment (ϵ = η^2^ = 1.8). In this case, the diagonalization of the full Hamiltonian in eq 3 leads to nominally exact results that can be compared with those obtained in the exciton approximation. Results in Figure S11 show marginal differences that are ascribed to ultraexcitonic interaction terms (which are disregarded in the excitonic approximation, but enter the full Hamiltonian, written on the diabatic basis): the result confirms that ultraexcitonic terms are small, and they can be safely disregardered.

Finally, two effective vibrational coordinates are introduced for each dye, for a grand total of four vibrational coordinates, *q*_*A*,1_, *q*_*A*,2_, *q*_*B*,1_, and *q*_*B*,2_. The vibronic Hamiltonian for the dimer in the exciton approximation reads:
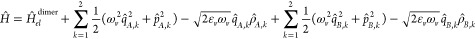
4where  is the Hamiltonian electronic part comprising mean-field and excitonic interaction terms with the respective screenings, *k* runs on the two molecular arms of each dye, and A and B refer to the first and second cyanine dye in Figure 4. The ionicity operators, , are diagonal on the diabatic basis, but they have off-diagonal elements on the exciton basis. Accordingly, vibronic couplings enter the picture mixing up degenerate and nondegenerate states.

The calculated spectra in Figure 5 are obtained with the same model parameters adopted to describe the monomer spectra (Table 3) and adjusting geometrical structure (caption of Figure 5) to best reproduce the OPA spectra. TPA spectra calculated with the same geometrical parameters are well in line with available experimental data.

**Figure 8:**
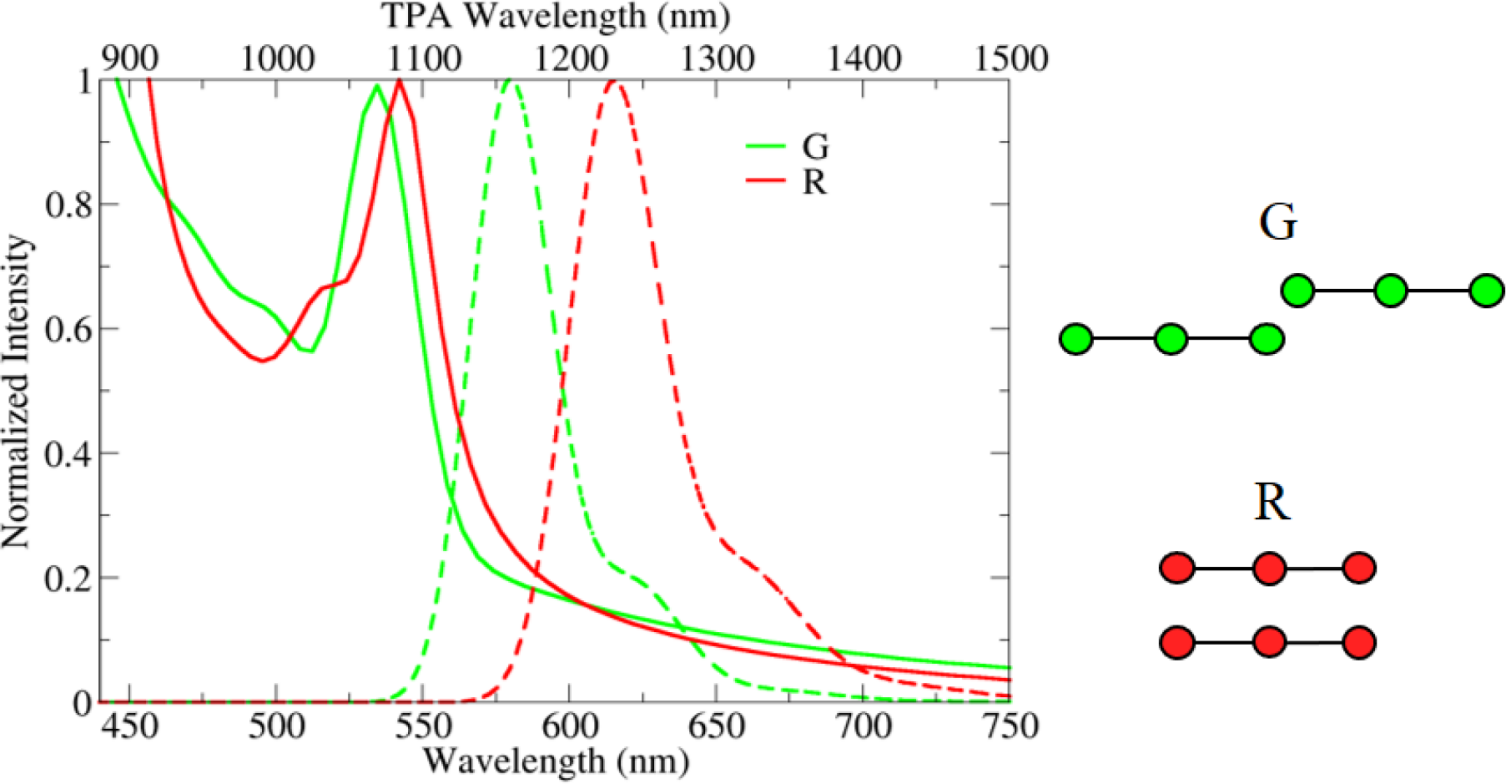
Emission (dashed lines) and TPA spectra (continuous lines) calculated to for two DiI dimers. Green spectra have *x* = 7 Å, *y* = 3 Å, *L* = 7 Å, while red spectra are relative to a cofacial dimer with *x* = 0 Å, *y* = 6.3 Å, *L* = 7 Å. Calculations are performed adopting static (ϵ) and optical (η^2^) dielectric constants of water, ϵ = 78 and η^2^ = 1.8, respectively.

Quite interestingly, the same model with the same model parameters reproduces well also the spectra collected in the hydrogel (Figure 8). The spectra of the green aggregate “G” in Figure 3 are reproduced adopting exactly the same model parameters adopted for DiI in Figure 5, while for the red aggregate “R”, the offset is set *x* = 0 and the interplanar distance *y* is increased, keeping all other parameters unaffected.

## Conclusion

In this work, a joint experimental and theoretical study of the optical properties of a family of commercial cyanine dyes (DiI, DiD, and DiR) and their aggregates in polar suspension is presented. The cyanine dyes under investigation differ only for the polymethinic bridge length (i.e., for the delocalization length). The bridge length affects the spectral position of OPA, emission, and TPA spectra of monomers in solution: the spectra move to the red if the length of the π-conjugated structure is increased. DiI, DiD, and DiR are good candidates as fluorescent probes for two-photon imaging, since TPA shows reasonable intensity in the OPA region. This band is nominally forbidden by symmetry, but it acquires intensity thanks to electron-vibration coupling. Experimental spectra of dyes in solution are fully rationalized by adopting the ESM approach developed some years ago for quadrupolar dyes.^31,33,34^

The tendency of cyanines to form aggregates is well-known.^14,15^ In the present work, aggregates of DiI, DiD, and DiR are prepared in water/ethanol mixtures and show different optical properties depending on the monomer. DiI clearly forms J-aggregates: the aggregates are emissive, their absorption spectrum is red-shifted compared to the monomer, the Stokes shift is negligible, and the ratio between the intensities of the 0–0 and 0–1 vibronic transitions both in absorption and in emission is increased with respect to the monomer. For DiI aggregates, we were able to collect TPA spectra, that, being slightly blue-shifted with respect to OPA, suggest a centrosymmetric structure for the aggregate. On the opposite, DiD and DiR form H aggregates, with a broad OPA band, slightly blue-shifted compared to the monomer, while the emission (not detectable for DiR aggregates) is very weak and red-shifted.^41^

The formation of different types (H or J) of aggregates for the different dyes can be ascribed to a delicate balance between the Coulomb repulsion of positively charged molecules and the hydrophobic effect. If we consider a dimer, only accounting for the hydrophobic effect, then the favored geometry would be a cofacial stacking in order to form the most extended possible hydrophobic pocket. In this geometry, however, positive charges would be perfectly superimposed, leading to large Coulomb repulsions. As a consequence, the dyes arrange themselves in a staggered geometry, the mutual shift being larger for shorter cyanines, where electrostatic repulsions are larger. This leads to the formation of almost cofacial H-aggregates for long cyanines such as DiD and DiR^19^ and to J-aggregates for the shorter DiI.

The proposed theoretical approach, based on essential-state models and accounting for molecular vibrations, allows us to rationalize in an excellent way the spectral properties of aggregates. To the best of our knowledge, this work is the first attempt to calculate nonlinear optical spectra of dimers of cyanines, accounting for molecular vibrations. Molecular vibrations play a crucial role on the spectral properties of centrosymmetric dyes where forbidden transitions acquire intensity as a result of Herzberg–Teller vibronic coupling.^60^

The effects of intermolecular interactions on the spectral properties of aggregates are particularly interesting. Accounting for intermolecular interactions is a critical issue, as two different screening regimes must be considered, one governed by the static dielectric constant and one governed by the dielectric constant at optical frequencies (i.e., the squared refractive index). This delicate conundrum is faced here based on the separation of the interaction occurring in the ground state (mean-field approach) and in the excited states (excitonic model). This issue is particularly relevant for polar solvents, in which these two numbers are considerably different.

To conclude, DiI, DiD, and DiR show very interesting linear and nonlinear spectral properties both in the monomeric form and in their aggregate form. The adopted theoretical approach, based on essential-state models, accounting for vibrational coupling, and for a detailed description of screening effects of intermolecular interactions, allows us to effectively reproduce linear and nonlinear optical spectra of aggregates of charged centrosymmetric dyes. The approach can be extended to supramolecular assemblies of polar or multipolar chromophores.

## Supporting Information Available

Physicochemical characterization of dyes in solution and of aggregates, details on essential-states modeling (PDF)
