# Supplementary material for: Aggregates of Cyanine Dyes: When Molecular Vibrations and Electrostatic Screening Make the Difference
Source: J Phys Chem C Nanomater Interfaces. 2023 May 18;127(21):10185–96. doi: 10.1021/acs.jpcc.3c01253 (PMC10240496; doi:10.1021/acs.jpcc.3c01253)
Supplement: Supplementary file 1 — jp3c01253_si_001.pdf [file jp3c01253_si_001.pdf]

Aggregates of Cyanine Dyes: when Molecular Vibrations and Electrostatic Screening Make the Difference

Francesco Bertocchi, Andrea Delledonne, Guillem Vargas-Nadal, Francesca Terenziani, Anna Painelli, Cristina Sissa*

Dipartimento di Scienze Chimiche, della Vita e della Sostenibilità Ambientale, Università di Parma, Parco Area delle Scienze 17A, 43124, Parma, Italy

Email: cristina.sissa@unipr.it

1 Physicochemical characterization

1.1 Cyanine dyes in solution

Table S1: Fluorescence average lifetimes of cyanine monomers in ethanol. [a]From Ref [1].

Cyanine	$\langle\tau\rangle$ (ns)
DiI	0.40[a]
DiD	1.31[a]
DiR	1.22

Table S2: Details of the bi-exponential fit of the fluorescence decay of DiR in ethanol.

Cyanine	τ_1 (ns)	B_1	τ_2 (ns)	B_2	χ^2
DiR	1.11	0.0108	5.94	4.5×10^{-5}	1.19

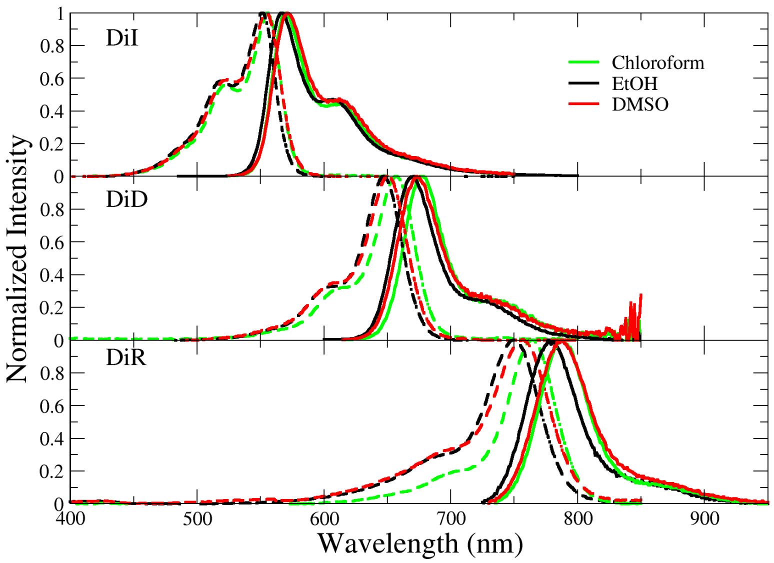

Figure S1: Normalized absorption (dashed lines) and emission (continuous lines) spectra of DiI, DiD and DiR in solvents of different polarity.

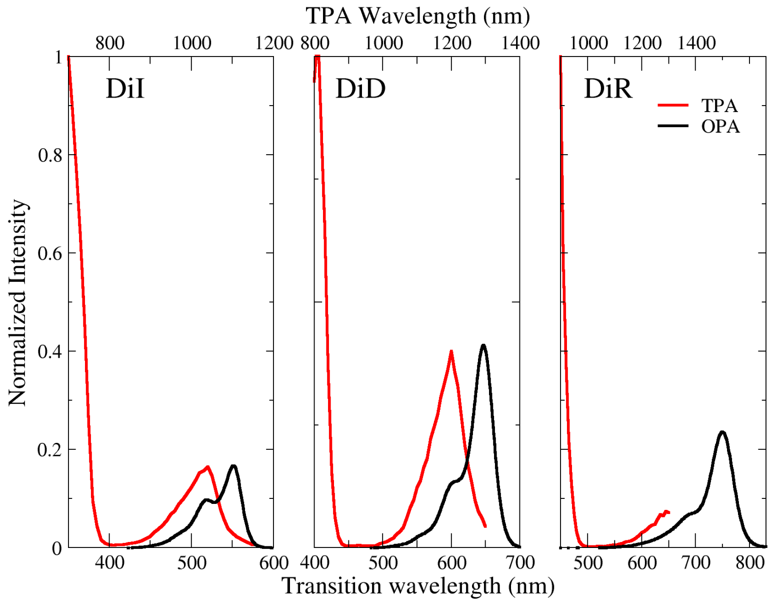

Figure S2: One-Photon Absorption (OPA, black lines) and Two-Photon Absorption (TPA, red lines) spectra of DiI, DiD and DiR in ethanol, including the rising edge of the TPA-allowed band.

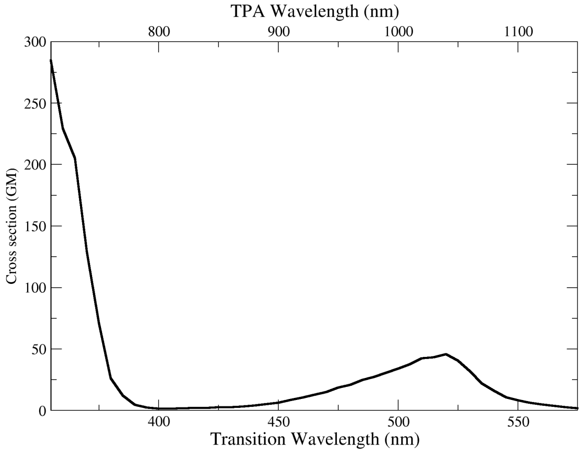

Figure S3: TPA cross section of DiI in ethanol as a function of wavelength, expressed in Goeppert-Mayer (GM) units.

1.2 Cyanine aggregates

Table S3: DLS data relative to DiI aggregates in water/ethanol 70/30 (m/m) and DiD, DiR aggregates in water/ethanol 90/10 (m/m). PdI indicates the polidispersity index of the suspension. The mean sizes of the peaks refer to the intensity distributions. Data were collected three hours after preparation, at 25°C and averaged over 3 measurements.

Aggregates	Z-average (nm)	PdI	Peak 1 mean size (nm)	Peak 1 intensity area %	Peak 1 volume area %	Peak 2 mean size (nm)	Peak 2 intensity area %	Peak 2 volume area %
DiI 70-30	83.1	0.461	120	89.6	6.1	14.1	6.8	93.4
St. Dev %	2.73	4.48	13.5	2.42	30.5	11.4	14	1.76
DiD 90-10	37.8	0.234	42.2	96.6	99.2	/	/	/
St. Dev %	6.32	11.2	10.6	3.1	0.7	/	/	/
DiR 90-10	72.8	0.327	85.7	93.2	22.4	18.8	6.8	77.6
St. Dev %	1.54	5.83	3.17	1.16	12.5	3.76	15.9	3.61

Hydrodynamic diameters expressed as average size values (Z-averages) or as the mean values of each peak were evaluated from intensity size distributions. Since the intensity distributions amplify the amount of larger particles, volume distributions have been reported to better appreciate the presence of smaller aggregates in the DiI and DiR suspensions (Fig. S4). The relative areas of the two peaks in the volume distributions clearly show that most of the aggregates belongs to the population with smaller size.

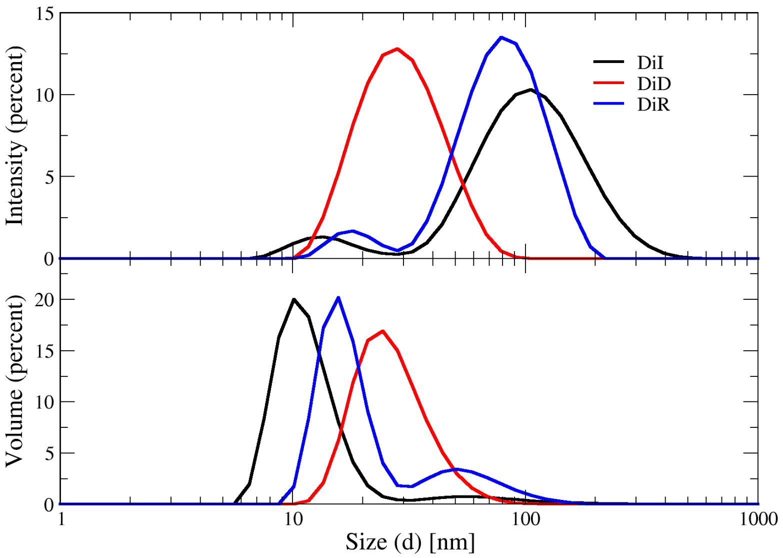

Figure S4: Size distributions by intensity (top panel) and by volume (bottom panel) of DiI, DiD and DiR aggregates obtained three hours after preparation as the average of three measurements each. Measurements were performed at 25°C.

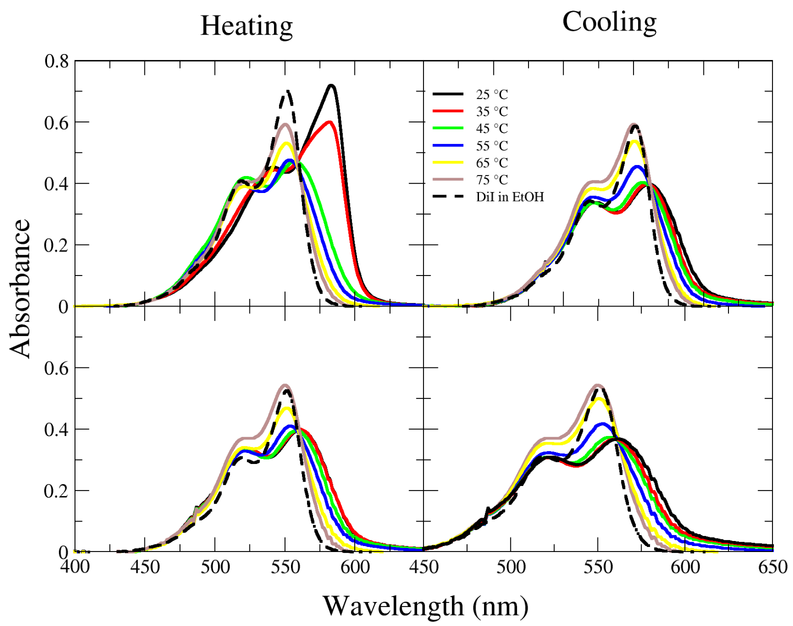

Figure S5: Upper panel: first cycle of heating (left) and cooling (right) of DiI aggregates in water/ethanol 70/30 (m/m). Lower panel: second cycle of heating (left) and cooling (right) of DiI aggregates in water/ethanol 70/30 (m/m).

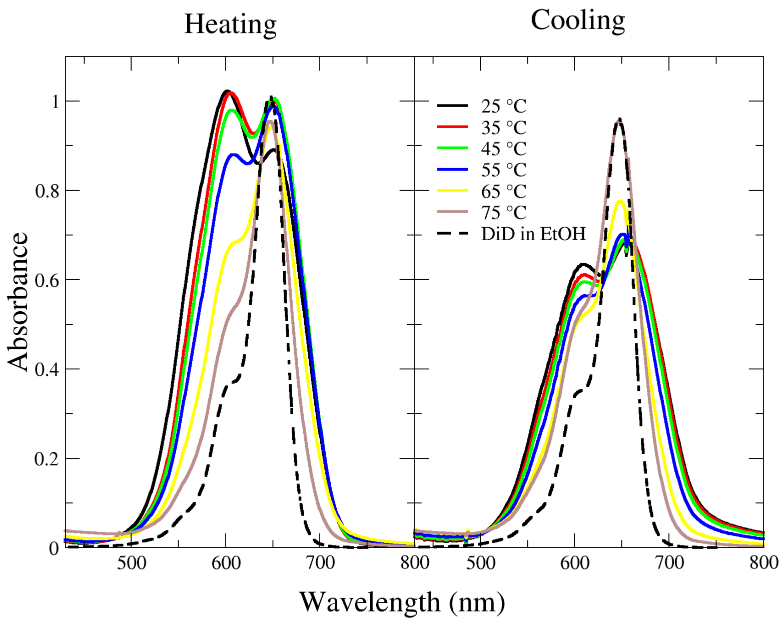

Figure S6: Left: heating of DiD aggregates in water/ethanol 90/10 (m/m). Right: cooling of DiD water/ethanol 90/10 m/m aggregates

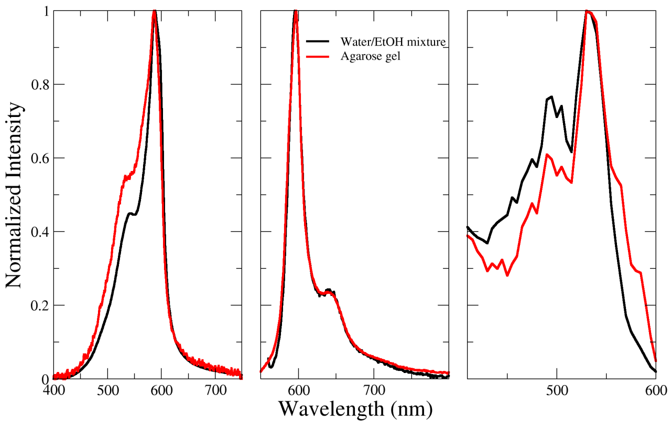

Figure S7: From right to left OPA, emission and TPA spectra of DiI aggregates suspended in the water/ethanol mixture (black line) and in the agarose gel (red line).

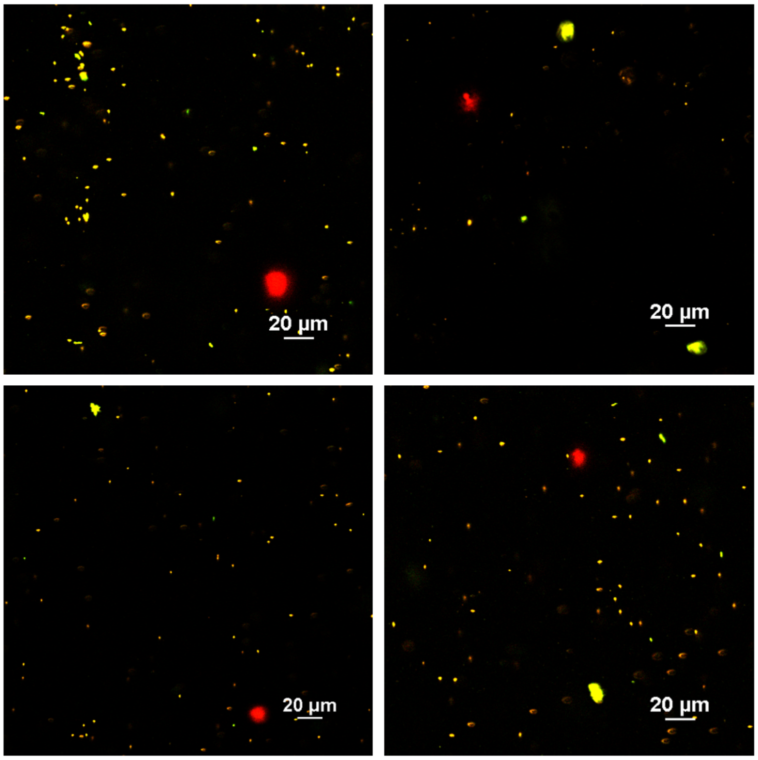

Figure S8: Two-photon excited fluorescence images acquired from DiI aggregates dispersed in a 2% agarose gel, obtained in different regions of the gel employed to acquire single-aggregate spectra.

2 Essential-state modelling

2.1 Three state model for cyanine dyes

The electronic Hamiltonian written on the diabatic basis ($|N\rangle$, $|Z_1\rangle$, $|Z_2\rangle$) for a cyanine reads:

$$\hat{H}_{el} = \begin{pmatrix} 0 & -\sqrt{2}t & -\sqrt{2}t \\ -\sqrt{2}t & 2z & 0 \\ -\sqrt{2}t & 0 & 2z \end{pmatrix} \tag{1}$$

Introducing the auxiliary operators:

$$\hat{\sigma} = \begin{pmatrix} 0 & 1 & 1 \\ 1 & 0 & 0 \\ 1 & 0 & 0 \end{pmatrix} \tag{2}$$

$$\hat{\rho}_1 = \begin{pmatrix} 0 & 0 & 0 \\ 0 & 1 & 0 \\ 0 & 0 & 0 \end{pmatrix} \tag{3}$$

$$\hat{\rho}_2 = \begin{pmatrix} 0 & 0 & 0 \\ 0 & 0 & 0 \\ 0 & 0 & 1 \end{pmatrix} \tag{4}$$

the Hamiltonian may be written as:

$$\hat{H}_{el} = 2z(\hat{\rho}_1 + \hat{\rho}_2) - \sqrt{2}t\hat{\sigma} \tag{5}$$

where $\hat{\rho}_1$ and $\hat{\rho}_2$ measure the charge on sites 1 and 2 of the cyanine dye (see Fig. 4 of the main text).

For a symmetric molecule, the expectation values of $\hat{\rho}_1$ and $\hat{\rho}_2$ are the same, and sum of their values, ρ, quantifies the charge displacement from the central site to each lateral unit, as shown in Fig. S9. ρ can be expressed as a function of model parameters z and t:

$$\rho = \frac{1}{2}\Big(1 - \frac{z}{\sqrt{z^2 + 4t^2}}\Big) \tag{6}$$

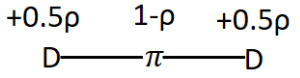

Figure S9: Charge distribution of the ground state.

The diagonalization of the electronic Hamiltonian yields three eigenstates, as linear combinations of the basis states:

$$\begin{aligned} |g\rangle &= \sqrt{1-\rho}\,|N\rangle + \sqrt{\rho}\Big(\frac{|Z_1\rangle + |Z_2\rangle}{\sqrt{2}}\Big) \\ |c\rangle &= \frac{1}{\sqrt{2}}(|Z_1\rangle - |Z_2\rangle) \\ |e\rangle &= \sqrt{\rho}\,|N\rangle - \sqrt{1-\rho}\Big(\frac{|Z_1\rangle + |Z_2\rangle}{\sqrt{2}}\Big) \end{aligned} \tag{7}$$

2.1.1 Coupling between electronic and vibrational degrees of freedom

The vibronic Hamiltonian of Eq. 2 in the main text can be rewritten using the bosonic creation and annihilation operators, $\hat{a}_i^\dagger$ and $\hat{a}_i$, respectively, associated to each vibrational mode:

$$\hat{H} = \hat{H}_{el} - \sqrt{\epsilon_v \omega_v}\sum_i \hat{\rho}_i(\hat{a}_i^\dagger + \hat{a}_i) + \omega_v \sum_i (\hat{a}_i^\dagger \hat{a}_i + \frac{1}{2}) \tag{8}$$

where $i = 1, 2$ runs over the two effective vibrational coordinates. The Hamiltonian is diagonalized on the basis obtained from the direct product of the 3 electronic diabatic states and the first N eigenstates of each harmonic oscillator. The dimension of the problem is $3N^2$, since we consider two effective vibrational coordinates. N is set to a large enough value to guarantee convergence; in our case $N = 10$ is a good choice.

The diagonalization of the Hamiltonian gives $3N^2$ numerically exact vibronic eigenstates. OPA spectra are calculated assigning a Gaussian bandshape to each vibronic transition (where the model parameter γ is the half width at half maximum of the Gaussian). The intensity of each vibronic transition is proportional to the squared transition dipole moment between the ground state and the relevant excited state. The spectrum is calculated summing up all the contributions of each vibronic transition. Emission spectra are calculated in the same way, but the transition dipole moments are calculated between the emissive state and all states having lower energy. The emissive state is chosen as the lowest-energy state having a sizable transition dipole moment from the ground state. For TPA spectra, the sum-over-state expression of third-order polarizability by Orr & Ward[2] is adopted. The detailed expressions for the calculation of spectra are reported in Ref [3].

2.2 Dimers of cyanines

2.2.1 The dimer basis set and explicit expression for the interactions V_{ij}

Table S4: Diagonal energies associated to each diabatic basis state for the dimeric structures.

Basis states	Diagonal energies
$\|\Phi_1\rangle$ ($\|NN\rangle$)	0
$\|\Phi_2\rangle$ ($\|NZ_1\rangle$)	$2z$
$\|\Phi_3\rangle$ ($\|NZ_2\rangle$)	$2z$
$\|\Phi_4\rangle$ ($\|Z_1N\rangle$)	$2z$
$\|\Phi_5\rangle$ ($\|Z_1Z_1\rangle$)	$4z$
$\|\Phi_6\rangle$ ($\|Z_1Z_2\rangle$)	$4z$
$\|\Phi_7\rangle$ ($\|Z_2N\rangle$)	$2z$
$\|\Phi_8\rangle$ ($\|Z_2Z_1\rangle$)	$4z$
$\|\Phi_9\rangle$ ($\|Z_2Z_2\rangle$)	$4z$

Eqs. 9 report interactions elements entering the dimer Hamiltonian in the diabatic basis, without screening due to the medium (geometrical parameters

x, y, L are defined in Fig. 4 of the main text).

$$\begin{aligned} V_{11} &= \frac{1}{4\pi\epsilon_0}\frac{1}{\sqrt{(x+L)^2+y^2}} \\ V_{12} = V_{21} &= \frac{1}{4\pi\epsilon_0}\frac{1}{\sqrt{x^2+y^2}} \\ V_{13} = V_{31} &= \frac{1}{4\pi\epsilon_0}\frac{1}{\sqrt{(x+\frac{L}{2})^2+y^2}} \\ V_{22} &= \frac{1}{4\pi\epsilon_0}\frac{1}{\sqrt{(L-x)^2+y^2}} \\ V_{23} = V_{32} &= \frac{1}{4\pi\epsilon_0}\frac{1}{\sqrt{(x-\frac{L}{2})^2+y^2}} \\ V_{33} &= \frac{1}{4\pi\epsilon_0}\frac{1}{\sqrt{x^2+y^2}} \end{aligned} \tag{9}$$

2.2.2 The mean-field problem

When two cyanines are close, electrostatic interactions become sizeable, and may lead to a redistribution of the charge within each molecule as to minimize the energy (mean-field effect). Since these charges are static, relevant interactions should be screened by the static dielectric constant of the medium, ϵ.

The mean-field problem is tackled in a self-consistent approach, as sketched in Fig. S10.

We consider centrosymmetric dimers, so that the two monomers in each dimer are equivalent. For aligned dimers ($x = 0$) the inversion center is conserved on each molecule, so that $\rho_1 = \rho_2$; but for staggered dimers ($x \neq 0$) the inversion symmetry of each dye is lost and $\rho_1 \neq \rho_2$. ρ_3 measures the amount of charge on the central site and of course $\rho_3 = 1 - \rho_1 - \rho_2$. The energies of diabatic states are renormalized by the interaction with the other cyanine.

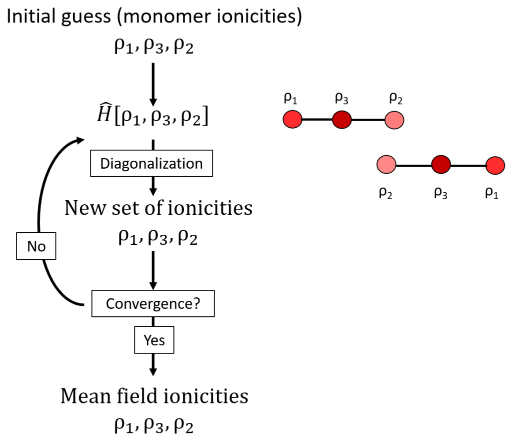

Figure S10: Self-consisting mean-field calculation scheme.

The mean-field energies of the diabatic states of each molecule depend on the charge on the sites of the other molecule, but, since the dimer is symmetric, we enforce equal charge distributions in the two molecules, ending up with a self-consistent problem. Specifically, the energies of the basis states of each dye in the mean field approximation read:

$$\langle N| \hat{H}_{el} |N\rangle = \frac{1}{4\pi\epsilon_0\epsilon}\left(\frac{\rho_2}{\sqrt{(x-\frac{L}{2})^2+y^2}} + \frac{\rho_3}{\sqrt{x^2+y^2}} + \frac{\rho_1}{\sqrt{(x+\frac{L}{2})^2+y^2}}\right) \quad (10)$$

$$\langle Z_1| \hat{H}_{el} |Z_1\rangle = 2z + \frac{1}{4\pi\epsilon_0\epsilon}\left(\frac{\rho_2}{\sqrt{x^2+y^2}} + \frac{\rho_3}{\sqrt{(x+\frac{L}{2})^2+y^2}} + \frac{\rho_1}{\sqrt{(x+L)^2+y^2}}\right)$$

$$\langle Z_2| \hat{H}_{el} |Z_2\rangle = 2z + \frac{1}{4\pi\epsilon_0\epsilon}\left(\frac{\rho_2}{\sqrt{(L-x)^2+y^2}} + \frac{\rho_3}{\sqrt{(x+\frac{L}{2})^2+y^2}} + \frac{\rho_1}{\sqrt{x^2+y^2}}\right)$$

We address the self-consistent problem by solving first the electronic problem for an isolated monomer, in order to get three initial values for the ground state ionicities ρ_1, ρ_2, ρ_3 (of course, $\rho_1 + \rho_2 + \rho_3 = 1$). These values are then inserted in a molecular Hamiltonian where the diagonal energies are corrected as in Eq. 10. The diagonalization of this Hamiltonian leads to new eigenvectors and hence to new ionicities, that are again inserted in Eqs. 10 to define a novel (improved) mean-field Hamiltonian. The cycle is repeated until ionicities at the n-th iteration differ from ionicities at the $(n-1)$-th iteration by the seventh decimal digit.

Results listed in Table S5 were obtained setting the dielectric constant to the pure water value, $\epsilon = 78$ (results do not vary appreciably for dielectric constant $\epsilon > 40$). Specifically, for the two systems, the table lists the charge residing on the three molecular sites, calculated for the isolated monomer and for the aligned and staggered dimers (of course for the monomer as well as for the

aligned dimer, $\rho_1 = \rho_2$). Due to the large screening of electrostatic interactions, the variation of charges is very small. In all systems, including the staggered dimer, the system maintains an overall centrosymmetric structure.

Table S5: Ground state charge distribution resulting from mean-field calculations on cyanine dimers setting the dielectric constant to the pure water value $\epsilon = 78$ (results are unaffected for smaller values down to ~ 40). Results refer to aligned ($x = 0$) or staggered ($x = 6$ Å) geometries for intermolecular distance $y = 4$ Å. The chromophore length is set to $L = 7$, 8 and 9 Å for DiI, DiD and DiR, respectively.

		ρ_1	ρ_3	ρ_2
DiI	Monomer	0.239	0.522	0.239
	aligned	0.239	0.521	0.239
	staggered	0.241	0.522	0.237
DiD	Monomer	0.236	0.529	0.236
	aligned	0.237	0.527	0.237
	staggered	0.238	0.528	0.234
DiR	Monomer	0.227	0.546	0.227
	aligned	0.228	0.545	0.228
	staggered	0.230	0.546	0.225

Table S6 reports the values of ground-state charge distributions for a medium-polarity environment ($\epsilon = 5$). Lowering the screening constant, leads to sizable mean-field effects, as expected.

Table S6: Ground state charge distribution resulting from mean field calculations on cyanine dimers setting the dielectric constant to $\epsilon = 5$. Results refer to aligned ($x = 0$) or staggered ($x = 6$ Å) geometries for intermolecular distance y= 4 Å. The chromophore length is set to $L = 7$, 8 and 9 Å for DiI, DiD and DiR, respectively.

		ρ_1	ρ_3	ρ_2
DiI	Monomer	0.239	0.522	0.239
	aligned	0.244	0.512	0.244
	staggered	0.266	0.524	0.210
DiD	Monomer	0.236	0.528	0.236
	aligned	0.243	0.514	0.243
	staggered	0.270	0.527	0.203
DiR	Monomer	0.227	0.546	0.227
	aligned	0.237	0.526	0.237
	staggered	0.268	0.543	0.189

2.2.3 Rotation from diabatic basis to exciton basis

Table S7: Energies associated to the excitonic basis states.

Wave functions	mean-field energies
$\|\Psi_1\rangle$ ($\|gg\rangle$)	0
$\|\Psi_2\rangle$ ($\|gc\rangle$)	E_c
$\|\Psi_3\rangle$ ($\|ge\rangle$)	E_e
$\|\Psi_4\rangle$ ($\|cg\rangle$)	E_c
$\|\Psi_5\rangle$ ($\|cc\rangle$)	$2E_c$
$\|\Psi_6\rangle$ ($\|ce\rangle$)	$E_c + E_e$
$\|\Psi_7\rangle$ ($\|eg\rangle$)	E_e
$\|\Psi_8\rangle$ ($\|ec\rangle$)	E_c+E_e
$\|\Psi_9\rangle$ ($\|ee\rangle$)	$2E_e$

The Hamiltonian in Eq. 3 of the main text includes both ground-state and excited-states interactions. Since ground-state interactions, screened by ϵ, are accounted for in the mean-field part, we now introduce interactions between excited states, and we screen them with η^2. To tackle this issue, we must rotate the basis from the initial diabatic basis to the excitonic basis (Tab. S7), i.e. the basis of the states derived as direct product of the three eigenstates, $|g\rangle, |c\rangle$ and $|e\rangle$ obtained for each dye in the mean field approximation. On this new basis, exciton interactions enter as off-diagonal elements between states having the same energy, and it is easy to single them out.

The eigenstates matrix ($\hat{U}$) of an interacting dimer at the mean field level is the rotation matrix which rotates the diabatic basis into the exciton basis. The matrix $\hat{U}$ is exploited to numerically rotate the basis and to rewrite relevant operators.

In the framework of the exciton approximation, we consider, out of the rotated interaction matrix, only the interaction terms that mix degenerate states, and specifically: $\langle gc| \hat{H} |cg\rangle, \langle ge| \hat{H} |eg\rangle$ and $\langle ce| \hat{H} |ec\rangle$.

From a purely electronic point of view, these matrix elements represent the interactions between transition charge densities: the $\langle ge| \hat{H} |eg\rangle$ element would be zero in the point dipole approximation, and it is small (but finite) in the extended dipole approach we adopted. Coupling with molecular vibrations could eventually modify the coupling between basis states, as discussed in the main text.

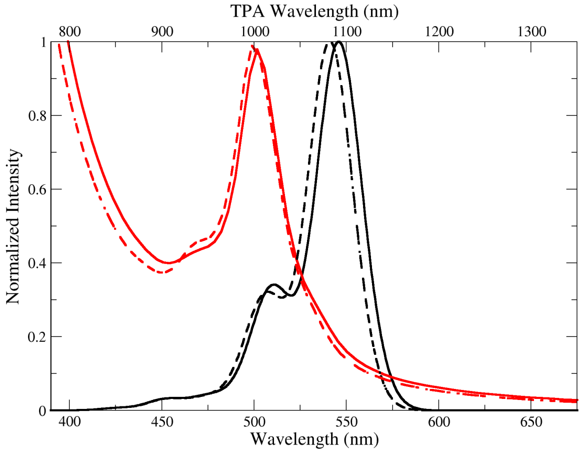

Figure S11: Continuous lines: OPA (black) and TPA (red) spectra calculated for the geometry chosen for the DiI dimer ($x = 7$ Å, $y = 3$ Å), using a diabatic basis in which all the interactions are screened by $\eta^2 = 1.8$. Dashed lines: OPA (black) and TPA (red) spectra calculated for the same geometry, considering a mean-field problem and exciton interactions screened by $\epsilon = \eta^2 = 1.8$. Calculations are performed accounting for electron-vibration couplings. The difference between dashed and continuous lines is due to the electronic ultraexcitonic terms, that are accounted for in continuous line spectra and are disregarded in dashed line spectra. The results confirm that ultraexcitonic terms have small contributions.

References

[1] J. Morla-Folch, G. Vargas-Nadal, T. Zhao, C. Sissa, A. Ardizzone, S. Kurhuzenkau, M. Köber, M. Uddin, A. Painelli, J. Veciana, K. Belfield, and N. Ventosa, "Dye-loaded quatsomes exhibiting fret as nanoprobes for bioimaging," *ACS Applied Materials & Interfaces*, vol. 12, p. 20253, 04 2020.

[2] B. Orr and J. Ward, "Perturbation theory of the non-linear optical polarization of an isolated system," *Molecular Physics - MOL PHYS*, vol. 20, pp. 513–526, 03 1971.

[3] C. Sissa, P. M. Jahani, Z. G. Soos, and A. Painelli, "Essential state model for two-photon absorption spectra of polymethine dyes," *ChemPhysChem*, vol. 13, no. 11, pp. 2795–2800, 2012.
